# A microbiological assessment of the oral hygiene of 24-72-month-old kindergarten children and disinfection of their toothbrushes

**DOI:** 10.1186/1472-6831-14-94

**Published:** 2014-08-02

**Authors:** Tahsin Celepkolu, İsmet Rezani Toptancı, Pakize Gamze Erten Bucaktepe, Velat Sen, Mehmet Sinan Dogan, Veysel Kars, Hamza Aslanhan, Ilknur Aslan, Tuba Dal, Ismail Yıldız, Yılmaz Palancı

**Affiliations:** 1 Department of Family Medicine, Dicle University Medical Faculty, 21280 Diyarbakir, Turkey; 2 Department Pediatric Dentistry, Dicle University Dentistry Faculty, Diyarbakir, Turkey; 3 Department of Pediatrics, Dicle University Medical Faculty, Diyarbakir, Turkey; 4 District State Hospital, Mazgirt, Tunceli, Turkey; 5 Department of Medical Microbiology, Dicle University Medical Faculty, Diyarbakir, Turkey; 6 Department of Biostatistics, Dicle University Medical Faculty, Diyarbakir, Turkey; 7 Department of Public Health, Dicle University Medical Faculty, Diyarbakir, Turkey

**Keywords:** Children’s dental health, Disinfection, Brushing teeth, Chlorhexidine

## Abstract

**Background:**

The objective of this study is to assess the index of decayed, missing and filled teeth (DMF-T), habit of brushing teeth, and the microbiological agents accumulating on the children’s toothbrushes for 4 weeks and response of these agents to disinfection via a chlorhexidine solution, then compare those results with the education and income levels of the children’s parents.

**Method:**

Included in the study were 187 children (96 in the control group and 91 in the experiment group – chlorhexidine) chosen randomly from 600 kindergarten children whose ages ranged from 24 months to 72 months. The children selected had not taken any antibiotics, antimicotics for three months and dental treatments during this trial. The distribution of these children to the groups was also done randomly. After performing a survey for the education, occupation, and income status of the parents, the children were examined and the number of decayed teeth was recorded. The children were given toothbrushes, toothpaste (with fluroide), and the solutions (including distilled water and chlorhexidine) for four weeks under the condition that toothbrushes were returned at the end of each week. The 14 different microbiological agents observed as a result of the assessment of the samples taken in the first week were also included in the assessments of the samples taken over the four-week period.

**Results:**

The decrease in the DMF-T index was found to be meaningful according to the differences in education, income, and occupation status of the parents. Of all the samples taken from the toothbrushes, the bacteria with the greatest rate of reproduction included *Streptococcus mutans, Escherichia Coli, Pseudomonas aeuroginosa, Enterococcus spp, Staphylococcus epidermidis* and *Candida albicans*. Except for Candida albicans, the other microorganisms taken as samples from the toothbrushes reproduced less overall. In the group using the solution with chlorhexidine, a meaningful decrease in bacterial reproduction was discovered compared to the control group.

**Conclusion:**

The findings of this study show that the education, occupation, and socioeconomic situations of the parents should be considered when discussing children’s oral and dental health. Moreover, the study shows that disinfection of toothbrushes in order to prevent reinfection and contamination oral flora with the bacteria again is important in terms of preventive medicine and family-children health.

## Background

Children usually go to a kindergarten, begin to socialize, come into contact with the environment more often, and show cognitive and sensual development at the age of 24–72 months. Due to the developing physical and psycho-emotional structures as well as socio-cultural environment, children participate in various new activities [1], and as a result, happen to be open to diseases they may come in contact with in the environment. From 24–72 months, children's nutritional habits change as well [2], which can cause children to suffer from problems resulting from poor nutritional habits, such as decayed teeth [3]. Because children at this age are actively and rapidly learning as they come in contact with the enviroment, it is important to introduce the habit of brushing teeth [1,4].

Toothbrushes come in varied qualities and are sterilized after packing following fabrication [5,6]. The first brushing is usually done between 30 seconds and 4 minutes of opening the toothbrush package; bacteria [6,7], viruses [5,8], and fungi [9,10] start to accumulate on the surface of the toothbrush bristles during that first brushing. Some microorganisms can stay alive on the toothbrushes from 24 hours to 7 days [5,6,11,12]. As a result of the routine usage of the toothbrushes, these microorganisms spread to the oral cavity of the same person over a period of time or to different people [13]. In general, toothbrushes of different family members are kept in the same place. Contamination occurs as a consequence of the contact of these toothbrushes, which can cause these microorganisms to spread from one individual to another. Contamination can easily spread outside the home to other children; not only are kindergarten children brushing their teeth in the same lavatory as friends and keeping toothbrushes in school lockers and schoolbags, but they are also sharing and exchanging toothbrushes, which causes microorganisms to transfer from one individual to the other. For this reason, disinfection of the toothbrushes has importance in terms of general health. Considering the importance of the subject, studies in the literature on this matter are insufficent [8,12,14].

The objective of this study is to assess the index of decayed teeth, missing teeth, and filled teeth, habit of brushing teeth, and the microbiological agents accumulating on the children’s toothbrushes as well as the reactions of these agents to disinfection via chlorhexidine solution, then compare those results to the education and income levels of the children’s parents.

## Methods

This study is both cross-sectional descriptive and prospective interventional study. Included in the study were 200 children (100 in the control group and 100 in the experiment group – chlorhexidine-) aged 24–72 months chosen randomly from 600 kindergarten children. Children had not taken any antibiotics, antimicotics, or dental treatments for three months. The control group (C) was comprised of 100 children given solution with distilled water. The other 100 children were accepted as experiment group (CHX) and given solutions with chlorhexidine. The distribution of these children was done randomly. However, during the study, a total of 13 children were excluded; 8 of them started antibiotic treatment because of an infection and 5 of them were absentees. In total, 187 children were involved into the study. Of 187 children, 92 were girls (49%) and 95 were boys (51%); 96 of these children (52%) constitute C group, and 91 (48%) constitute CHX group.

The parents of the children who were accepted to participate in to the study were informed about the study and their signed approvals were obtained.

The children involved were given extra attention whether they had got all of their baby teeth or not and the ones who did not complete all of them were not included into the study. After giving enough information to the parents about the study, the children and the parents were taught how to brush teeth efficiently by dentists. After performing a survey about the occupation, education, and income status of the parents, oral examinations of the children were completed. Children were given travel type, soft toothbrushes with caps and no extra features. The children were given toothbrushes, solutions (with distilled water and chlorhexidine), and toothpaste (including fluroide) under the condition that toothbrushes would have to be returned at the end of each week. The children were instructed to brush their teeth at least twice a day for at least two minutes under the control of their parents and teachers. The spray boxes given to the patients (50 cc) were numbered; some had distilled water (forming the C group) and the others had 0.12% chlorhexidine gluconate (forming CHX group). The children were instructed to wash their toothbrushes after each brushing by applying the solution given by parents and kindergarten teachers onto every surface of the toothbrush.

On the first day of the study, after giving information and performing examinations, the children were given the toothbrushes (by coding), toothpaste, and solution boxes (distilled water or chlorhexidine). After a week, the toothbrushes were collected, stored by providing cold chain at 2–8°C and brought to microbiology laboratory. After taking the first toothbrushes, the second toothbrushes were distributed according to study group; these procedures were repeated for four weeks. At the end of the fourth week, the toothbrushes were taken back and the children were given a new toothbrush as a present.

Culturing of the toothbrushes was done in Microbiology Laboratory of Faculty of Medicine at Dicle University. The bristles of the toothbrushes were immersed in broth (with thioglycollate (Oxoid, UK) in order to identify the microorganisms, then were incubated in an incubator at 35 ± 2°C for 24 hours. After incubation, liquid food-lot 5% sheep blood agar (Oxoid, UK), EosinMethilene Blue agar (Oxoid, UK) were inoculated, and they were incubated in an incubator at 35 ± 2°C aerobically for 20–24 hours. The conventional methods from the food-lots on which bacteria breeding were detected and VITEK 2 (bioMérieux, Marcyl'Etoile, France) were identifed in terms of bacteria with full automatic microbiology system. For S. mutans analysis, it was incubated in the enriched form of Basitracin-Sukkuroz food-lot with Jensen and Brattall modification and kept under 37°C for 4 days in order to observe reproduction.

The study was approved at the Ethics Committee of Dicle University on 25^th^ November 2011 by the number 312 decision.

### Statistical analysis

IBM SPSS 15.0 for Windows statistics packet programme was used for the statistical analysis of our research data. The measuremental variables were presented by average ± standard deviation (SD). Categoric variables were presented by numbers and percentages (%). For the consecutive measurements, Variance Analysis was applied for the reproductive progress in the 1st, 2nd, 3rd, and 4th weeks of microorganisms in the control (C) and Chlorhexidine (CHX) groups. Friedman Test was used in order to compare the reproductive progress of microorganisms in C and CHX groups separately in the 1st, 2nd, 3rd, and 4th weeks. In the same way, Wilcoxon test was used in order to compare the microorganisms in C and CHX groups separately each week. Chi-square (χ2) test analysis was used in order to compare the qualitative variables between groups. The hypotheses were two-sided and the results p ≤ 0.05 were accepted as statistically significant.

## Results

A total of 187 children were included in our study assessment and the average age was 48.13 months (±8.74). The average number of decayed teeth per child was 3.74 (±3.55), and they did not take any treatments for these teeth.

Of 187 children, 92 were girls and 95 were boys. The average age of the girls was 48.72 months (±9.28). The average age of the boys was 47.56 months (±8.20). There was no statistically significant difference (p = 0.366). When comparing the number of the decayed teeth between the girls and boys, it was observed that the number of decayed teeth was less in the girls than in the boys. However, this difference was not statistically significant (p = 0.064). There was no statistically significant difference between the boys’ and the girls’ family income status, though the parents of the boys were observationally more wealthy than the girls’ parents (p = 0.230).

Considering all of the children, there was a significant difference in the number of decayed teeth between the children whose parents both worked and the children who only had one working parent (p = 0.001).

It was seen that the index of the decayed teeth decreased as the education status of the fathers got higher (p = 0.001). Whereas average number of decayed teeth was 6.28 (±4.01) in the children whose mother earned a degree of secondary school and less, this rate regressed to 3.05 (±1.08) in the children whose mothers were educated (p = 0.001). A decrease in the incidence of decayed teeth was observed in the children whose mothers earned a high school degree and whose fathers earned a university degree (p = 0.001).

Of 187 children, 96 children were accepted as control group (C) and 91 children were accepted as chlorhexidine (CHX) group. They were given distilled water (for C group) and chlorhexidine water (for CHX group) for their toothbrushes as a single cure. In the control group (C), average age was 48.41 months (±8.79). Average age was 47.84 months (±8.74) in the CHX group. The age difference between two groups was not significant. In the oral examinations before the study, DMF-T index was 3.28 (±3.28) in C group and 4.22 (±3.77) in CHX group. Although DMF-T index was higher in H group, this difference was not significant (p = 0.070). Regarding the income status, there was no significant difference between the groups (p = 0.567).

In the microbiological studies, 14 microorganisms reproduced in the first samples taken from the toothbrushes used by the children both in C and H groups. These microorganisms were also taken into consideration when analyzing the samples obtained over the course of the study. These microroganisms were Streptococcus mutans, Escherichia Coli, Staphylococcus epidermidis, Neisseria spp, Bacillus spp., Pseudomonas aeuroginosa, Acinetobacter spp., Staphylococcus auerus, Enterococcus spp., Proteus spp., Pantoea agglomerans, Candida albicans, Klebsiella pneumonia, and Enterobacter spp. Of all of these microroganisms, the samples with the greatest reproductive rate in the microbiological cultures were Streptococcus mutans, Escherichia Coli, Pseudomonas aeuroginosa, Enterococcus spp, Staphylococcus epidermidis, Candida albicans. Since the others reproduced only in small amounts on the toothbrushes, they were not included in the assessment.

These microbiological agents were seen in the samples taken from the children in both C group and CHX group. Table 1 shows the reproductive rate for each microorganism, each week and whether this reproduction was significant.

**Table 1:** The number of bacteria to breed toothbrush for 4 weeks and significance levels

	**1st week**		**2nd week**		**3rd week**		**4th week**		**p**^**1**^	**p**^**2**^	**p**^**3**^
	**C(n = 96)**	**CHX(n = 91)**	**C(n = 96)**	**CHX(n = 91)**	**C(n = 96)**	**CHX(n = 91)**	**C(n = 96)**	**CHX(n = 91)**			
*Streptococcus mutans*	89 (93%)	42 (46%)	72 (75%)	25 (28%)	66 (69%)	19 (21%)	57 (57%)	8 (9%)	0.000	0.000	0.000
*Escherichia coli*	78 (81%)	30 (33%)	41 (43%)	16 (18%)	36 (38%)	13 (%14)	34 (35%)	8 (9%)	0.000	0.000	0.000
*Staphylococcus epidermidis*	51 (53%)	25 (28%)	10 (10%)	13 (14%)	9 (9%)	5 (6%)	5 (5%)	7 (8%)	0.037	0.000	0.000
*Pseudomonas aeruginosa*	41 (43%)	23 (25%)	30 (31%)	16 (18%)	3 (3%)	5 (6%)	34 (35%)	6 (7%)	0.000	0.304	0.000
*Enterococcus faecalis*	13 (14%)	11 (12%)	10 (10%)	5 (6%)	9 (9%)	4 (4%)	12 (13%)	2 (2%)	0.044	0.699	0.022
*Candida albicans*	17 (18%)	6 (7%)	0 (0%)	3 (3%)	0 (0%)	2 (2%)	0 (0%)	2 (2%)	0.542	0.000	0.323

p^1^ = The possibility in the comparison of breeding microorganisms on the toothbrushes in the control and chlorhexidine groups.

p^2^ = The possibility in the comparison of breeding microorganisms on the toothbrushes only in the control group.

p^3^ = The possibility in the comparison of breeding microorganisms only in the chlorhexidine group.

(P < 0.005 was accepted significant).

The bacteria breeding on the toothbrushes of C and CHX groups were also assessed each week and the results are shown in Table 2.

**Table 2:** Weekly relevance level of the breeding bacteria in the C and CHX groups (p)

**Week**	**Streptococcus mutans**	**Escherichia coli**	**Staphylococcus epidermidis**	**Pseudomonas aeruginosa**	**Enterococcus faecalis**	**Candida albicans**
1-2	0.000	0.000	0.000	0.027	0.092	0.000
1-3	0.000	0.000	0.000	0.000	0.021	0.000
1-4	0.000	0.000	0.000	0.006	0.052	0.000
2-3	0.123	0.364	0.087	0.147	0.672	0.648
2-4	0.000	0.067	0.055	0.392	0.805	0.648
3-4	0.004	0.351	0.708	0.501	0.834	1.0

**Figure 1:**
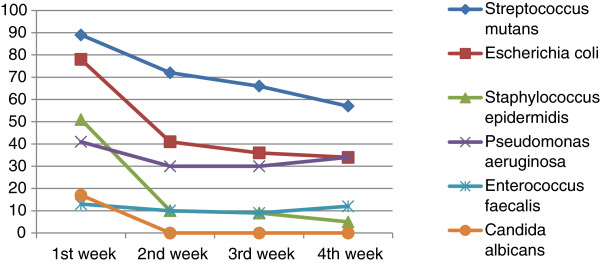
Bacteria in breeding toothbrushes only in group C for 4 weeks.

**Figure 2:**
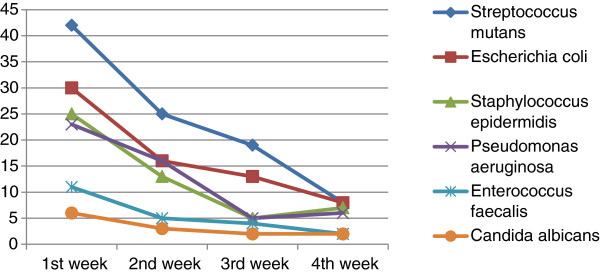
Bacteria in breeding toothbrushes only in group CHX for 4 weeks.

The differences in reproduction by week for microorganisms on the toothbrushes of C group are shown in Table 3. The differences in reproduction by week for microorganisms on the toothbrushes of CHX group are shown in Table 4. The last two cases are shown respectively in Figures 1 and 2.

**Table 3:** Weekly relevance level of the breeding bacteria only in the C group (p)

**Week**	**Streptococcus mutans**	**Escherichia coli**	**Staphylococcus epidermidis**	**Pseudomonas aeruginosa**	**Enterococcus faecalis**	**Candida albicans**
1-2	0.000	0.000	0.000	0.086	0.467	0.000
1-3	0.000	0.000	0.000	0.093	0.248	0.000
1-4	0.000	0.000	0.000	0.345	0.796	0.000
2-3	0.273	0.475	0.763	1.0	0.782	1.0
2-4	0.007	0.286	0.197	0.527	0.593	1.0
3-4	0.095	0.752	0.285	0.546	0.004	1.0

**Table 4:** Weekly relevance level of the breeding bacteria only in the CHX group (p)

**Week**	**Streptococcus mutans**	**Escherichia coli**	**Staphylococcus epidermidis**	**Pseudomonas aeruginosa**	**Enterococcus faecalis**	**Candida albicans**
1-2	0.008	0.016	0.019	0.162	0.083	0.317
1-3	0.000	0.003	0.000	0.000	0.035	0.102
1-4	0.000	0.000	0.001	0.001	0.013	0.157
2-3	0.273	0.564	0.059	0.008	0.739	0.655
2-4	0.001	0.102	0.157	0.012	0.257	0.655
3-4	0.016	0.225	0.527	0.739	0.257	1.0

## Discussion

The first goal of this study was to make the children involved gain the habit of brushing their teeth daily, and to create a conscious control mechanism of the children at home by the parents and at kindergarten by teachers. It was stated in the literature that the incidence of decayed teeth was lower in the individuals who gained the habit of brushing teeth at an early age [15,16]. The influence of the education given to the parents and the children can be followed through the weekly changes of bacteria rates taken from the the toothbrush samples. At this age, it is highly important to educate children on hygiene and encourage good habits against pathogens coming from outer environment with changing nutritional habits [2], socializing [1], and changing environmental conditions. Likewise, it was seen in some studies from Europe that family help when brushing teeth has an important role in order to detect childhood decayed teeth early and prevent them [4,17]. However, it was observed in a different study that it does not play a significant role in the United States of America [18].

Average DMF-T index of 187 children involved in this study was 3.73, which is higher than the other age groups involved in other similar studies. In a study perfomed by Chu and his colleagues in 2012, they reported that average DMF-T for 764 children who were at the pre-school term was 2,2 [15]. DMF-T was 1.6 for the 5-year-old children included in a 2003 study in the UK [15]. In a study from Australia, the average DMF-T was 1.5 [19]. In a study from Brazil in 2012, the DMF-T index of preschool children was 1.53 ± 2.63 [20].

In our study, there was not a statistical significance between income levels of the parents and number of decayed teeth, which is the similar results in the previous studies [4,17].

It was seen in our study that the children whose parents' education levels were low had more decayed teeth than the ones whose parents had university and academic education. It was also stated in a study made by Chu and his colleagues in 2012 that the rate of the decayed teeth was higher in children whose parents came from low socio-economic backgrounds and had low education levels [15].

The disinfection of a new toothbrush should be done in order to prevent the formation of bacterial biofilm layer on the toothbrush blisters after the first brush [5]. Moreover, the disinfection of the toothbrushes should be done daily, and toothbrushes should be changed every 3–4 months [21,22]. In many studies, a variety of methods were used for the disinfection of toothbrushes including microwave, boiled water, ultraviolet light, and chemical agents, in particular chlorhexidine-gluconate solutions [23]. Other researches attempted to determine a new method for disinfection using chlorhexidine or other materials with disinfectant characteristics to cover the surfaces of toothbrushes [24]. In this study, MS colony number decreased significantly in the group given chlorhexidine-gluconate spray (0,12%) compared to the control group. This result is parallel to the studies by Filho and his colleagues in 2000 and 2006 [5,6]. In our study, a significant decrease was observed in the analysis made for C.albicans concerning the control and chlorhexidine groups. We think that it results from both regular teeth-brushing and the influence of chlorhexidine-gluconate. Similar results were obtained using a commercial solution with 0.12% CHX in a study by Nascimento and his colleagues [9]. We believe that the colonization of the other bacteria results from the influence of CHX. We are of the opinion that decreases in MS colonization in the oral environment could occur with regular teeth brushing, to some extent.

## Conclusion

Independent from the income and education states of the parents, brushing teeth is very important from 24–72 months (kindergarten) children. It was observed that the rates of microorganism reproduction decreased after brushing teeth regularly, considering the reproduction rates of the microorganisms were very high in the samples taken at the end of the first week. A significant decrease was seen in the rate of accumulating bacteria on the toothbrushes with the use of chlorhexidine-gluconate solution (0.12%) for disinfection. Children ages 2–6 are the most important age groups for developmental and behavioral learnings. This age group is also very important in order to protect public health. It will be appropriate to care about children's oral and dental health and, apart from a family health doctor and a pediatrician, a dentist should accompany the kindergarten health examinations for early diagnosis and treatment. We are of the opinion that making children brush their teeth more efficiently for oral and dental health as well as protecting toothbrushes microbiologically is very important for children's dental health and accordingly public health. We believe that more studies on this subject should be performed.

## Competing interests

The authors declare that they have no competing interests.

## Authors’ contributions

We certify that all individuals listed as authors of this manuscript: 1) have made substantial contributions to conception and design, or acquisition of data, or analysis and interpretation of data; 2) have been involved in drafting the manuscript or revising it critically for important intellectual content; 3) have given final approval of the version to be published; and 4) agree to be accountable for all aspects of the work in ensuring that questions related to the accuracy or integrity of any part of the work are appropriately investigated and resolved. TC conceived and designed the study and mostly wrote the manuscript. IRT contributed to the conception and design of the study and has made children’s oral examination as well as assisted writing the manuscript. PGEB made the interpretation of the data, and assisted with drafting and writing the manuscript. VS contributed to the conception and design of the study, assisted with analysis and interpretation of the data. MSD assisted children’s oral examination and writing the manuscript. VK contributed to the conception and design of the study, assisted with analysis and interpretation of the data. HA assisted with analysis and interpretation of the data, and assisted with drafting and writing the manuscript. IA assisted with analysis and interpretation of the data, and assisted with drafting and writing the manuscript. TD has made microbiological cultivation of toothbrushes in the laboratory and assisted with drafting and writing the manuscript. IY assisted with analysis and interpretation of the data, and assisted with drafting and writing the manuscript. YP contributed to the conception and design of the study, assisted with analysis and interpretation of the data, and assisted with drafting and writing the manuscript.

## Pre-publication history

The pre-publication history for this paper can be accessed here:

http://www.biomedcentral.com/1472-6831/14/94/prepub
